# The impact of continuous lenalidomide maintenance treatment on people living with multiple myeloma—a single-centre, qualitative service evaluation study

**DOI:** 10.1007/s00520-024-08663-4

**Published:** 2024-07-02

**Authors:** Caroline Buck, Francisco Brenes Castillo, Elena Bettio, Joanne Land, Orla McCourt, Helen Poole, Rachel Tarling, Kwee Yong, Rakesh Popat, Lydia Lee, Annabel McMillan, Xenofon Papanikolaou, Ke Xu, Chara Kyriakou, Neil Rabin, Ashu Wechalekar, Abigail Fisher, Jonathan Sive

**Affiliations:** 1 Institute of Epidemiology and Health, University College London, London, UK; 2 Haematology Department, University College London Hospital, London, UK; 3 Research Department of Haematology, UCL Cancer Institute, University College London, London, UK; 4 School of Psychology, Liverpool John Moores University, Liverpool, UK

**Keywords:** Multiple Myeloma, Lenalidomide, Maintenance, Qualitative

## Abstract

**Purpose:**

Continuous lenalidomide maintenance treatment after autologous stem cell transplantation delivers improvement in progression free and overall survival among newly diagnosed multiple myeloma patients and has been the standard of care in the UK since March 2021. However, there is scant information about its impact on patients’ day-to-day lives. This service evaluation aimed to qualitatively assess patients receiving lenalidomide treatment at a cancer centre in London, in order that the service might better align with needs and expectations of patients.

**Methods:**

We conducted 20 semi-structured interviews among myeloma patients who were on continuous lenalidomide maintenance treatment at a specialist cancer centre in London. Members of the clinical team identified potentially eligible participants to take part, and convenience sampling was used to select 10 male and 10 female patients, median age of 58 (range, 45–71). The median treatment duration was 11 months (range, 1–60 months). Participants were qualitatively interviewed following the same semi-structured interview guide, which was designed to explore patient experience and insights of lenalidomide. Reflexive thematic analysis was used for data analysis.

**Results:**

Four overarching themes were as follows: (i) lenalidomide: understanding its role and rationale; (ii) reframing the loss of a treatment-free period to a return to normal life; (iii) the reality of being on lenalidomide: balancing hopes with hurdles; (iv) gratitude and grievances: exploring mixed perceptions of care and communication. Results will be used to enhance clinical services by tailoring communication to better meet patients’ preferences when making treatment decisions.

**Conclusion:**

This study highlights that most patients feel gratitude for being offered continuous lenalidomide and perceive it as alleviating some fears concerning relapse. It reveals variations in side effects in different age groups; younger patients reported no/negligible side effects, whilst several older patients with comorbidities described significant symptom burden, occasionally leading to treatment discontinuation which caused distress at the perceived loss of prolonged remission. Future research should prioritise understanding the unique needs of younger patients living with multiple myeloma.

**Supplementary Information:**

The online version contains supplementary material available at 10.1007/s00520-024-08663-4.

## Background

Multiple myeloma (MM) is an incurable malignancy of plasma cells in the bone marrow and is the second most common haematological malignancy, with incidence set to rise due to an ageing population [1, 2]. Patients with MM often experience high symptom burden including fatigue, bone pain, fractures, and kidney failure [3].

In the UK, initial treatments for MM include high-dose chemotherapy and autologous stem cell transplantation (ASCT) [4], which can cause side effects such as neuropathy, fatigue, and gastrointestinal issues [5]. An older patient profile means that age-related comorbidities often coincide with these symptoms [6]. The MM disease trajectory is characterised by periods of active disease followed by treatment-induced remission that shorten as the disease progresses, eventually becoming non-responsive to treatment [7–9], causing uncertainty for many patients [10]. A meta-aggregation of 11 qualitative studies examining experiences of MM suggests that grief and isolation are common [11], with quantitative surveys suggesting that patients can experience depression [12], anxiety [13], and poor health-related quality of life (QoL) [14, 15].

The outcomes of patients with MM have improved with novel therapies in the last 20 years [16]; however, relapse is almost inevitable; thus a key treatment objective is to prolong progression-free survival (PFS) and overall survival (OS) whilst minimising toxicity [17]. In 2021 the National Institute of Clinical Excellence (NICE) approved continuous lenalidomide for eligible UK National Health Service (NHS) patients receiving ASCT post-induction chemotherapy [18], after randomised trials demonstrated extended PFS and OS compared to placebo by enhancing the depth of disease response through the suppression of residual malignant cells [19–23]. Patient-reported outcomes from an observational study of 169 patients receiving lenalidomide maintenance and 137 receiving no maintenance suggest manageable side effects [24]. However, clinical trial data demonstrate that neutropenia, fatigue, neuropathy, and gastrointestinal disturbances are common, particularly in the first 6 months of treatment [20, 21], with two large trials indicating that 29% of participants experienced severe enough side effects to discontinue treatment [19]. Data also show that lenalidomide increases the risk of secondary malignancies [25].

Before lenalidomide maintenance was used in this setting, individuals would typically enter a treatment-free phase after ASCT, where they might experience a reasonable QoL without treatment burden [26], but maintenance eliminates that opportunity. A discrete choice experiment examining treatment preferences suggested that medication breaks provide an opportunity for patients to detach from the ‘illness’ experience [27], a finding echoed in a qualitative evaluation of ASCT during the COVID-19 pandemic [28]. However, a survey of 736 MM patients exploring perceptions of maintenance versus side-effect burden showed two-thirds would opt for maintenance if it offered PFS benefit, even if it was mildly toxic and showed no OS improvement [29]. Whilst the PFS and OS benefits of lenalidomide are proven, both short- and longer-term toxicities reported in trials mean that the trade-off between efficacy and harm should be considered [30] Yet to our knowledge, no exploration of patients’ experience of being on continuous lenalidomide has been conducted, and a deeper understanding of how it might impact individuals’ QoL is scant. Qualitative research can explore the complexity of human experiences, providing insights into individuals’ perspectives [31, 32]. The objectives of this qualitative service evaluation at a single-centre department in London were to:

1. Explore patients’ understanding of the role of lenalidomide in their treatment for MM.
2. Examine patients’ experience of lenalidomide, including perceived impact on QoL and experience of side effects.

It was anticipated that the findings would be consolidated and presented to clinicians so that improvements could be integrated into future care, through the delivery of communication better suited to patients’ needs.

## Methods

### Participants and recruitment

Participants were patients in a specialist cancer centre in a large, university-affiliated central London hospital, where they were receiving MM treatment. As this was a study exploring experiences of patients on lenalidomide maintenance treatment, participants were eligible for the study if they had undergone induction chemotherapy followed by ASCT and were receiving continuous lenalidomide. If participants were unable to give informed consent or did not possess adequate proficiency in the English language, they were deemed ineligible to take part. Patients were approached by members of the clinical care team and asked to consider taking part in an interview examining the perceived impact of lenalidomide on their lives. Participants were recruited using convenience sampling, where the selection was based on their availability and willingness to contribute. The invitation letter stated there was no obligation to participate, and that non-participation would not impact ongoing care. If they agreed to take part, they were emailed the study information and given a week to consider participation. In line with Health Research Authority guidelines, ethical approval was not required as the study was a service evaluation [33]. Those who agreed to participate gave written, informed consent to the research team contacting them to arrange interviews, and to their details being stored on a secure system called the University College London (UCL) Data Safe Haven. A target sample size of 16–24 participants was determined as acceptable for achieving adequate information power, with the understanding that more would be recruited if this was not attained [34].

### Data collection

One-to-one in-depth semi-structured qualitative interviews (mean duration 52 min) were conducted (via telephone/MS Teams) between June and October 2022. Three health psychology researchers (2 females: CB and EB, 1 male: FB) with qualitative research experience carried out the interviews and took notes. One researcher (CB) had previously conducted research among MM patients [28]. A semi-structured guide was designed by the team to examine patients’ experiences of being on lenalidomide, their understanding of its role, and its perceived impact on their lives (See Supplementary Material 1 for guide). The guide was pilot tested on one patient by CB to ensure flow, and to ensure it encouraged participants to share experiences of lenalidomide and how information was understood/interpreted. The final sample size was based on the concept of ‘information power’ [34], which suggests that study aims, data richness, and analytical strategy dictate sample size. According to the guidelines of this commonly used paradigm in qualitative research, the sample size was deemed adequate when the incorporation of additional data contributed minimal or negligible alteration to the findings, in this case after 20 interviews had been conducted. Interviews were audio-recorded, anonymised, and transcribed verbatim by an external transcription service with a UCL data sharing agreement, with identifiable information removed. The researchers had no pre-existing relationship with participants.

### Analysis

Interview transcripts were analysed using reflexive thematic analysis (RTA) [35], which acknowledges researcher subjectivity in data engagement [36]. Analysis was underpinned by a critical realist ontology that recognised how participants’ experiences were influenced by their social, cultural, and personal beliefs [37]. The epistemological stance taken was one of social constructionism, which assumes knowledge is co-constructed by researchers and participants [38].

Two researchers (CB and FB) adhered to RTA’s six stages [39]. Microsoft Word and Excel were used to manage the data. In *Data familiarisation*, analytical points of interest were identified by reviewing transcripts; *Coding* involved noting relevant segments and applying labels; *Generating initial themes* identified shared patterns that formed the basis for developing themes; *Developing and reviewing themes* ensured themes communicated a compelling story; *Refining, defining and naming themes* assigned descriptions and names to themes; *Writing up* involved completion of the analysis. HP and RT reviewed themes with the lead author (CB) to minimise bias and promote reflexivity, and CB kept a reflexive journal throughout the process [35]. The Consolidated Criteria for Reporting Qualitative Research (COREQ) checklist was the basis for the reporting of this research [40] (See Supplementary Material 2).

## Results

### Participants

Twenty-four patients were approached and 20 agreed to take part. Two declined because of ill health and two did not give reasons. The mean interview duration was 52 min (range 22 to 98 min). See Table 1. for participant characteristics.

**Table 1.** Participant characteristics (*n* = 20)

	Median (range)
Age	58 (45–71)
Time (months) since diagnosis	31 (15–90)
Time (months) on lenalidomide	11 (1–60)
Gender Male:Female	10:10
Ethnic group	
Black African	1
Black Caribbean	2
Other black background	1
Asian Indian	1
White British	12
Other ethnic group	1
Unknown	2

### Themes and subthemes

Four themes and 12 subthemes were developed from the data; these are presented in Table 2 with illustrative quotes. Overarching themes were (1) lenalidomide: understanding its role and rationale, (2) reframing the loss of a treatment-free period to a return to normal life, (3) the reality of being on lenalidomide: balancing hopes with hurdles, and (4) gratitude and grievances: exploring patients’ mixed perceptions of care and communication.

**Table 2:** Main themes, subthemes and quotes from the data set. From: The impact of continuous lenalidomide maintenance treatment on patients living with Multiple Myeloma—a single-centre, qualitative service evaluation study

Themes	Subthemes	Illustrative quotes	
1. Lenalidomide: understanding its role and rational	1.1 Attitudes towards lenalidomide seemed to depend on timing of diagnosis	*I was prepared. I knew that I would have to take medication forever, you know, to maintain the situation … any medicine wouldn’t come as a surprise because I was expecting to be on some kind of maintenance medication. (ID 9, Female, 51)*	*I thought I was just going to, you know, gradually get off all the meds … it’s what helped get me through the stem cell transplant … I was just a bit, you know, deflated. (ID13, Female, 66)*
	1.2 “*Lenalidomide doubles remission*” is convincing	*With the research that was done, it has doubled the length of the remission. So, you know, I felt very thrilled that I was offered it on the National Health because it’s quite expensive. (ID10, Female, 61)*	*I remember he was saying the average, and he’s always said, the average after stem cell is two and a half years. The average with Len can be five and a half years. (ID15, Female, 59)*
2. Reframing the loss of a treatment-free period to a return to normal life	2.1 Lenalidomide: offering a welcomed sense of security	*It makes me feel a bit safer like … I don’t have to look over my shoulder quite so much because there is this sort of tiny little safety net in place. So that’s got to be positive. (ID10, Female, 61)*	*My fear is relapse … and I know that I’m doing something about it to stop a relapse so that gives me confidence. (ID3, Female, 68)*
	2.2 Maintenance perceived as ‘just another pill’ versus a demanding treatment	*It’s absolutely easy. Nothing to be worried about or anxious about. It kind of blends into your life …it’s part of life… you wake up in the morning and you brush your teeth in the same way. (ID7, Male, 55)*	*I just call it taking my tablets … I'm a patient for all of 10 s, you know what I mean … swallow it down and you know then I get on with my life again. (ID19, Male, 52)*
	2.3 Participants experienced strong emotions in their transition from frontline to maintenance treatment	*Well you know, “be happy now,” and it’s like well yes, changing my mindset took longer I think and people were expecting me to be jumping around with joy, you know you’ve come through the transplant and you’re onto the next stage … you’ve got the freedom now to do what you want. (ID5, Female, 71)*	*And yes, now it’s a little bit different, it’s a little bit, it’s like you know, go and live your life for a bit and, which is a little bit hard …took me a while to get used to I think (ID11, Male, 48)*
3. The reality of being on lenalidomide maintenance: balancing hopes with hurdles	3.1 Differing assumptions about impact of lenalidomide: from ambivalence to miracle cure	*Somebody could be on no maintenance at all and have longer remission than somebody on the maintenance. (ID11, Male, 48)*	*If I’m really honest I would like to think that I’m going to be fine. The average is five and a half but I’m going to be one of those that get 10. And then I’ll do it again. (ID10, Female, 61)*
	3.2 Impact of side effects on patients	*It’s very unobtrusive, and it’s very straightforward, and yes, it doesn’t impact on my life really. Yes [laughs]- all I need to do is remember to take a pill. (ID2, Male, 59)*	*The fitness and the fatigue, clearly, is with me all the time. I would think twice about going on a very active holiday … so yes, it has changed my viewpoint and made me realise that there are things that I am not going to be able to regularly do or enjoy. (ID4, Male, 70)*
	3.3 Blood tests as the focal point of worry and hope	*I’m very interested in my bloods … I’m very interested to check how it’s doing, I don’t just leave it for somebody to tell me they’re all right … I look compared to the normal … you have to laugh at me because I really don’t know anything but I’ve got pages and pages of the stuff … I just want to know. (ID20, Female, 70)*	*Every time you have a blood test you think, oh right, thank God, I missed it that time until the next time. But you know, touch wood, but it seems to be going in the right direction. So fingers crossed. (ID15, Female, 59)*
4. Gratitude and grievances: exploring patients’ mixed perceptions of care and communication	4.1 Perceptions of care	*I cannot fault the professionalism, you know they’re wanting to help you, never in one part of it were they angry with you, they just wanted to help you, calm you to make sure that they explain everything before they did it. (ID8, Male, 63)*	*We all feel a bit intimidated when we are speaking to people such as consultants … I don’t believe I did ask enough questions … you are just accepting of what you are being told, you know? You bow down to people’s knowledge, particularly from a medical point of view. (ID, Male, 70)*
	4.2 Variable communication: seeking clarity about facts and side effects of lenalidomide	*I guess a doctor can’t always tell you how you’re going to feel, or what to expect, you know, because it’s all sort of second-hand information, isn’t it? It’s a world they don’t know about … I think I just benefit from speaking to people who you know had my sort of illness. (ID14, Male, 57)*	*It’s only from looking at and reading, you know, and hearing other stories that I see that people who have got other health conditions have said, “Well, when I started Len I started getting a lot of gastro problems,” and you know, “I went to A&E because it flared up and it’s only since I’ve been on Len.” So, you know, different things have happened to different people and they’re not sure whether it’s because of the Len. Some have had heart problems, “I get palpitations. I get breathless. (ID3, Female, 68)*
	4.3 Differing needs of younger patients	*More of a conversation rather than just a consultant telling you …it’s up to you to kind of ask questions …it’s explained to you that you’re entitled to have the conversation, you’re entitled to ask questions, you’re entitled to say you're sure now, it’s not ‘this is what you need and you’ve got no choice’, you’re allowed to kind of have a conversation about your own treatment because at the end of the day it is you that is having it (ID19, Male, 52)*	*On my 100 days after my second stem cell, I’m expecting to see Dr X, to give me my ‘yes we are quite happy with how you’re doing and this is where we are going to go’…on that appointment I saw another doctor, it completely threw me out, completely threw me out – I was…I don’t know, unsettled I think … it was a stranger, I’d had amongst 18 months of going through all that I’ve gone through … if I had a problem I called and it was wither Dr X or Y. And there I was sat in the room with a complete stranger … it unsettled me (ID1, Male, 56)*
	4.4 Patient dissatisfaction with remote medical consultations	*I actually saw a doctor face to face the last time. That was amazing. That hadn’t happened for months and months … it’s better than a telephone appointment I must say. I did feel happier. (ID13, Female, 66)*	*I think as a patient sometimes … you not only want to speak to a human being but you want to see a human being if that makes sense, do you know what I mean, you kind of want that person to … see you. (ID19, Male, 52)*

## Lenalidomide: understanding its role and rationale

### Attitudes towards lenalidomide seemed to depend on timing of diagnosis

A pattern in the narratives was noted, whereby patients’ attitudes towards taking lenalidomide seemed to vary based on the timing of the diagnosis. In addition, there appeared to be varied understanding and evaluation of maintenance treatment after initial discussion about lenalidomide with the medical team. Some patients diagnosed before NICE approval claimed to have been told that lenalidomide might impede normal life, as it precluded medication breaks. By contrast, it appears that among those diagnosed after NICE approval, many tended to feel that clinicians conveyed how lenalidomide could extend/improve their lives.

Many patients described how the challenges of their initial MM treatments such as ASCT often diverted attention from having the capacity to consider future treatments such as lenalidomide maintenance, leading to a failure to engage fully in discussions about it. This arguably added to variations in understanding about lenalidomide when it came time to start treatment. Furthermore, a small number of participants misunderstood what ‘maintenance’ actually meant, leading to more misunderstanding; some assumed it was an infrequent regime, e.g. an occasional scan or chemotherapy infusion rather than a long-term undertaking.

### ‘*Lenalidomide doubles remission*’ is convincing

Most patients came to believe that continuous lenalidomide would significantly extend remission and many were convinced that clinicians told them it would double their remission. This belief appeared to be decoded as a form of *certainty* of efficacy, which contrasted with the unpredictability of MM. Recognition of its considerable cost was apparent, alongside gratitude for its provision free of charge at point of delivery by the NHS.

## Reframing the loss of a treatment-free period to a return to normal life

### Lenalidomide: offering a welcomed sense of security

Participants describing resistance towards lenalidomide recalled a mindset change once they understood it offered an antidote to the uncertainty of waiting for relapse. Staving off relapse was associated with affording them the chance to feel ‘normal’ again, allowing time with family and friends, to pursue hobbies, or establish a previously eradicated sense of freedom. Consequently, the perceived disadvantages of lenalidomide were outweighed by optimism for these benefits.

### Maintenance perceived as ‘just another pill’ versus a demanding treatment

Participants highlighted that the lenalidomide regimen was manageable, especially in contrast to the demands of induction and ASCT. For many, taking one pill a day did not constitute ‘treatment’ so much as ‘swallowing a tablet’, often alongside others.

### Participants experienced strong emotions in their transition from frontline to maintenance treatment

Several participants reported how they surrendered to being a ‘patient’ when first diagnosed, ‘opting out’ of decision-making amid procedures/preparation for ASCT. In contrast, participants described that having to make choices again (i.e. decide whether to take lenalidomide) caused them consternation, which coincided with reduced doctor-patient interactions. Whilst desirable, having less medical support raised expectations from friends/family that life had returned to normal. Some patients described experiencing mental health symptoms (anxiety, depression), as they recognised implications of their illness and its perceived impact on their lives.

Several participants described a desire to address health behaviours, i.e. alcohol intake, diet, exercising, and stress in order to maximise treatment efficacy during maintenance. However, some (mostly older patients) expressed concerns about adopting new regimes without discussions with their medical team, which proved difficult due to time constraints. Some younger patients claimed to have sought health guidance from sources outside of the team to expand their health knowledge.

## The reality of being on lenalidomide maintenance: balancing hopes with hurdles

### Differing assumptions about the perceived impact of lenalidomide: from ambivalence to miracle cure

Patients' attitudes towards the perceived impact of lenalidomide on their disease trajectory appeared to fall into three groups: (i) a small group exhibited ambivalence, harbouring doubts regarding lenalidomide’s efficacy, often swayed by anecdotal evidence of people surviving with no additional treatment after ASCT; (ii) several participants appeared pragmatic, demonstrating an understanding of lenalidomide’s role in prolonging remission, whilst acknowledging that relapse was inevitable; (iii) a large group displayed ‘magical thinking’, affording lenalidomide with the capacity to provide them an exceptionally long remission and/or cure.

Despite differences in attitudes, the perceived benefits of lenalidomide appeared to strengthen for many over time, described as providing a conceptual safety net that kept the disease at bay. The desire to feel safe when evidence suggests precariousness was widespread. Some participants depicted lenalidomide as a buffer against the inevitability of relapse, a drawing of a metaphorical line in the sand.

### Perceived impact of side effects on patients

Three distinct groups emerged in this sample: (i) a predominantly younger group of participants experienced minimal /no side effects, e.g. mild fatigue and bloating; (ii) a mixed age group experienced more pronounced symptoms such as joint pain, gastrointestinal discomfort, and fatigue that were reported to impact daily life to a tolerable degree (two patients’ dosage was reduced to alleviate symptoms); (iii) a group of mainly older participants experienced side effects which were reported as markedly impacting QoL; rashes, fatigue, gastric disturbances, bone pain; two patients had to discontinue treatment due to debilitating symptoms.

Many participants recounted difficulty in distinguishing between lenalidomide’s side effects and residual symptoms from MM; age was also cited as a proxy for aches/pains. This ambiguity served to reinforce the decision to continue taking it. A few participants worried when they experienced *no* side effects, as it led them to assume lenalidomide was ineffective. Some participants admitted sporadically skipping doses to lessen side effects, not always admitting this to the medical team.

Concerns about toxicity/secondary cancers were latent, growing more recessive as patients tolerated lenalidomide and fears were diminished. A small number avoided mentioning side effects to clinicians for fear of being taken off lenalidomide. Those required to reduce dosage or discontinue expressed deep disappointment at the thought of lost therapeutic benefits.

### Blood tests as the focal point of worry and hope

Blood tests appeared to incite anxiety among many participants, as they were perceived as the only solid gauge of lenalidomide’s efficacy. For those with no or many side effects, test results provided evidence of the one thing patients knew was critical—continued remission as seen through paraprotein levels or light-chain analysis. Whilst most claimed faith in lenalidomide’s effectiveness, seeds of doubt emerged each time a blood test was due.

## Gratitude and Grievances: Exploring patients’ mixed perceptions of care and communication

### Perceptions of care

Most participants were positive about the MM department, and the perceived high standard of care received made several reticent to voice complaints for fear of causing offence. However, some described concerns about sudden changes in consultant and difficulties accessing the helpline.

### Variable communication: seeking clarity about facts and side effects of lenalidomide

Several participants mentioned a lack of sufficient opportunity to discuss lenalidomide during consultations. Perceptions regarding the provision of side-effect information varied, with some reporting adequacy whilst others perceived ambiguity from clinicians themselves. Several turned to Myeloma UK/Facebook groups for lived experience about lenalidomide that was felt beyond the medical team’s remit.

### Differing needs of younger patients

In this sample, patterns emerged in the narratives of patient needs that differed across age groups; younger patients (45–55 year olds) questioned and challenged the conventional doctor-patient paradigm, wanting a more balanced communication. They also sought detailed information about lenalidomide. Meanwhile, older participants mentioned that they mostly accepted what medics said without question. Continuity of care seemed crucial for all participants, but particularly so for younger males, as the medical team often served as their only outlet for discussing their illness. Moreover, younger participants expressed difficulty in finding MM support groups relatable, as they predominantly consisted of older individuals.

### Patient dissatisfaction with remote medical consultations

Whilst acknowledging the need to avoid in-person consultations during the COVID-19 pandemic, many expressed dissatisfaction that remote doctor-patient interactions persisted. Telephone consultations were often perceived as impersonal, limiting the ability to be seen and heard by doctors.

### Service evaluation

Findings were consolidated and presented to clinicians in a multidisciplinary team (MDT) meeting, with discussion around how they could integrate these into care (i.e. how they communicated information to patients who were going to begin lenalidomide maintenance). Main topics of interest for the clinicians were those presented in  the final theme.

## Discussion

This study found the timing of diagnosis impacted how maintenance was internalised by participants; consistent with other studies [26, 28], those diagnosed with pre-NICE approval were more likely to describe disappointment about missing treatment breaks. Conversely, later diagnosis reduced awareness of forfeiting a break in medication. Variations in understanding may have been compounded by participants’ distress at diagnosis, where shock hampered the ability to process information meaningfully, a finding noted in other studies [7].

Several participants reported how uncertainty about relapse adversely affected their lives, supporting findings from previous research [7]. Learning that lenalidomide can prolong PFS offered a semblance of certainty which juxtaposed with MM’s volatility. Furthermore, the act of taking one tablet was not perceived as ‘treatment’ per se, especially compared to induction chemotherapy/ASCT. Our study highlights that clinicians should aim to gradually disseminate information, highlighting that taking a daily pill offers the potential to prolong remission, thus deviating from the perception of it being a demanding regime.

In this sample, younger patients reported fewer side effects from lenalidomide treatment than older individuals, and a large mixed-age group viewed their symptoms as tolerable. These findings align with previous studies that showed the effects of lenalidomide are manageable [20, 21, 24]. However, a subset of predominantly older patients with comorbidities reported considerable symptom burden in this study, including digestive issues, bone pain, and fatigue, occasionally resulting in treatment discontinuation. Cessation of treatment due to adverse effects is reported in the literature, but with little detail [19–21, 24]. Whilst differences across ages were evident in this study, it is difficult to be conclusive, as evidence suggests the first 6 months of treatment are more likely to elicit side effects, and many participants in our sample had only recently started taking lenalidomide [20, 21]. Several participants were concerned about reducing/ missing doses, others worried if side effects did not materialise, and some underreported side effects for fear of being taken off it. These findings support a recent study that demonstrated reticence of some patients at describing side effects to their medical team [41], pointing towards a need to ensure patients understand the trade-off between efficacy and toxicity of lenalidomide, and for clinicians to encourage honest reporting of symptoms. Further research examining the impact of discontinuing maintenance treatment on patients would be a useful addition to the literature, specifically examining ways of supporting people who cannot tolerate it.

Several individuals conveyed experiences of strong negative emotions during transition from frontline to maintenance treatment, which is supported by research [12, 13]. Some expressed a desire to improve their health, aligning with studies demonstrating how MM patients assume the role of active consumers of medical information to inform self-care practices [7, 42]. These observations present an opportunity for the design/delivery of health behaviour interventions to help MM patients improve their well-being.

Whilst support groups clearly have a role, it is important to recognise their contribution to patient misinformation [43]. Whilst several patients held a realistic view of lenalidomide’s role, some recounted apocryphal tales of miraculous recoveries. Denial has been examined in previous research [44] showing that it constitutes a helpful coping strategy to navigate uncertainty [10]. Whilst denial can be dysfunctional, it may afford patients the space to absorb distressing information, and could have an adaptive role [45]. However, it does suggest a need to allocate adequate time/resources for patients to engage in discussions about lenalidomide to ensure clarity. This could include providing opportunities for pre-maintenance patients to interact with individuals further along in their treatment journey (moderated by a health professional), effectively fulfilling patients’ desire for lived experiences whilst reducing the spread of unsubstantiated information.

The qualitative literature on the experiences of younger MM patients is limited [46], and this study highlighted unique challenges faced by younger people who sought more inclusive communication, detailed information on lenalidomide, and continuity of care. It also emphasises the significance of personalised approaches and support services for managing MM in younger patients, highlighting the need for further exploration of this group to increase understanding.

Most participants trusted the care and expertise of healthcare staff in this MM department, but concerns arose over inconsistent information about lenalidomide. Criticisms of remote consultations’ cursory nature were also voiced. Studies on telemedicine barriers highlighted that phone/video calls reduce perceptions of emotional support [47, 48], suggesting that in-person consultations could enhance communication.

## Strengths and limitations

To our knowledge, this is the first qualitative exploration of the influence of lenalidomide on patients’ lives. Qualitative research provides depth and understanding of individuals’s lived experiences. Interviews in this study were conducted by researchers unconnected to MM clinical service, which may have minimised social desirability. Our study had limitations: sampling at one NHS MM department limits generalisability; group comparisons (i.e. between younger and older patients) are based solely on the authors’ interpretations and need further studies to confirm this; telephone/video call interviews may have overlooked non-verbal cues and rapport-building observed in person.

## Conclusions

Thanks to novel therapies MM has been transformed into a treatable disease with improving survival rates, yet it remains incurable. Patients might increasingly endure continuous medication to control the disease and prolong PFS and OS, whilst knowing that MM will eventually take its toll, and coming to terms with this can be challenging [49]. This study suggests that the promise of lenalidomide can sometimes cloud rational decision-making due to the intense desire for survival, leading to patients minimising side effects and experiencing anxiety about dosage and potential discontinuation of lenalidomide if they cannot tolerate it. Current knowledge about treatment effects is predominantly derived from clinical trials, and trial participants might not fully represent the broader MM population [50–52]. Findings provided key points for clinicians on how to personalise and improve service. Future studies could assess if changes were implemented to the service, and determine barriers and facilitators to change. This information could be implemented for a behavioural change intervention utilising the Integrated Promoting Action on Research Implementation in Health Services (i-PARIHS) framework [53]. Further qualitative research on the real-world symptom burden of treatments on patients’ lives could contribute to a more comprehensive understanding of the impact of MM, helping patients cope with the increasingly chronic nature of this disease.

### Supplementary Information

Below is the link to the electronic supplementary material.

Supplementary file1 (DOCX 23 KB)

Supplementary file2 (DOCX 69 KB)

## Data Availability

Can be made available on request.
