# Supplementary material for: The impact of continuous lenalidomide maintenance treatment on people living with multiple myeloma—a single-centre, qualitative service evaluation study
Source: Support Care Cancer. 2024 Jul 2;32(7):479. doi: 10.1007/s00520-024-08663-4 (PMC11219377; doi:10.1007/s00520-024-08663-4)
Supplement: Supplementary file 1 — Supplementary file1 (DOCX 23 KB) [file 520_2024_8663_MOESM1_ESM.docx]

**Interview schedule**

This guide helps the interviewer cover the key topics covering the aims of the study. However the interview should be based in a respondent-sensitive nature, to allow the researcher to be more flexible through the questions and to give the space to the participant to raise additional issues. Interviewers must take into consideration the sensitive subject of the interview and be careful not to push participants if they are distressed, but also make the best effort to prompt to understand the reasons for responses. Questions do not have to be asked in the order they are presented – the aim is for the interview to feel like a natural conversation. Some items in the interview may be covered in detail by responses to previous questions so don’t need to be repeated. If unsure they can be framed as “You may have already covered this…”

**Introduction**

*My name is [name] and I am a Health Psychology researcher working with the Myeloma Team. Thank you for agreeing to take part in this interview. These interviews are part of a clinical service evaluation designed to help the team understand more about how people with myeloma feel about the idea of being on continuous lenalidomide maintenance therapy (also known as Rev or Revlimid) after stem cell transplantation. Throughout this interview I will call this ‘len maintenance therapy’. My questions are only a guide, so please feel free to talk openly and honestly and tell me about any aspects of your treatment or situation that you think are important. If I ask anything that you don’t want to answer just tell me and we will move on. I am not a member of the myeloma clinical team, so I can’t answer any medical questions, but if you are worried about anything please talk to your clinical team.*

*Before we start can I just confirm you are happy for me to record this conversation?*

<ensure recording on>

*Now we will start the interview*

- Firstly, what made you decide to take part in this interview? *(warm up question)*
- How long have you been on len maintenance therapy?
- I am interested in understanding more about any discussions you had with the myeloma team about going on to len maintenance

- At what point after your diagnosis was this recommended to you?

- Did this come as a surprise to you? *(prompt on why or why not a surprise)*

- Did you feel you got all of the information you needed?  *(If not Prompt: What were the barriers that led to not receiving all the information needed? Find out if they feel they have all the information they need now and if so where this additional info came from)*

- Would you change anything about the way the information was given to you?  *(if yes prompt on what they would change and how they would have preferred considering both content and context e.g. in person vs online)*

*-* Is there anything more the myeloma team could have done/or could do to support you through this process?

- Did you seek advice or support from anyone outside the myeloma team? *(if yes ask from whom – e.g fellow myeloma patients, myeloma support groups, charities (Myeloma UK, Blood Cancer UK), other)*

- I would like to understand a bit more about **your** experience of being on len maintenance therapy:

-How has it made you feel? *(Prompt: physical symptoms, feelings, anxiety, stress)*

-Has it affected your day-to-day life in any way? *(if yes prompt on how)*

-Is it what you were expecting? *(prompt on whether feel better or worse than expected and where these expectations came from)*

- Imagine a set of scales where the positives of this treatment were on one side and negatives on the other. Describe how that looks to you *(prompt for the balancing of benefits versus downsides, as them to think about what’s on each side and also whether the scales are balanced, or tipped towards the positive or negative)*

-If you were to describe the experience of this treatment to someone with myeloma who was about to start, what would you say? What advice would you give them to help them feel prepared?

- As you know we are particularly interested in how people with myeloma feel about being on **continuous** len maintenance treatment (with no break).

- What benefits are you aware of, either now or in future?

*-* Do you have any idea how long these benefits might last? *(Prompt on understanding of duration of remission/prompt on sources of these beliefs)*

-Do you know of any negative aspects? *(Prompt on sources)*

- Do you have any idea what might happen next? *(Trying to get at understanding of what they think will happen if they relapse)*

- The myeloma team are keen to know what people understand about the phrases ‘depth of response’ and ‘minimal residual disease (or MRD) status’ – do these mean anything to you? *(if yes probe on where understanding came from e.g. discussion with team)*

- What would you do (or have you done) if you experienced treatment side effects or symptoms?

*Prompt: Have you communicated your symptoms/side effects (if any) to the clinical team? If yes, how was your experience communicating with the clinical team, if not why not?)*

*Prompt: Would you consider stopping the treatment in favour of a free-from-treatment period of the if the side effects became too much of a problem?*

- In future we would like to design a survey that could help us understand more about how people with myeloma feel about their treatment experience

- Tell me about any surveys you have been asked to fill in before about how you are feeling or how you have experienced treatment *(prompt on what they thought was good or bad)*

- What types of questions do you think we should ask to really understand how people with myeloma are affected by their experiences of treatment?

- Is there anything else you would like to tell us that we haven’t already discussed?

**Concluding guidelines**

a) Thank the participant for their time

b) Let them know they can contact you by email in the future for any possible additional comments or clarifications
